# The evolving role of family physicians during the coronavirus disease 2019 crisis: An appreciative reflection

**DOI:** 10.4102/phcfm.v12i1.2478

**Published:** 2020-06-09

**Authors:** Louis S. Jenkins, Klaus B. Von Pressentin, Kartik Naidoo, Rachel Schaefer

**Affiliations:** 1 Department of Family and Emergency Medicine, Division of Family Medicine and Primary Care, Faculty of Medicine and Health Sciences, Stellenbosch University, Cape Town, South Africa; 2 Department of Family and Emergency Medicine, George Regional Hospital, Garden Route, George, South Africa; 3 Primary Health Care Directorate, University of Cape Town, Cape Town, South Africa; 4 Mossel Bay Sub-District, Western Cape Department, South Africa; 5 Division of Family Medicine, School of Public Health and Family Medicine, Faculty of Health Sciences, University of Cape Town, Cape Town, South Africa

**Keywords:** family medicine, training, roles, community, COVID-19

## Abstract

Ten family physicians and family medicine registrars in a South African semi-rural training complex reflected on the coronavirus disease 2019 (COVID-19) crisis during their quarterly training complex meeting. The crisis has become the disruptor that is placing pressure on the traditional roles of the family physician. The importance of preventative and promotive care in a community-oriented approach, being a capacity builder and leading the health team as a consultant have assumed new meanings.

## Introduction

Ten family physicians and family medicine registrars are in their first quarterly training complex meeting for 2020. The setting is the Garden Route district in South Africa, a semi-rural area with a population of 620 000 people, of which 80% are dependent on six government hospitals for their health needs. The mood in the room is circumspect. Many questions hang in cyberspace. We can all see and hear each other, but there is a difference. For the first time since 2007 we are meeting via videoconferencing, all sitting in front of computers, spaced out over 120 km. Since our last physical meeting in 2019, the world has changed.

## The coronavirus disease 2019 pandemic

An invisible enemy, the novel coronavirus, has infected more than 1.9 million people globally, with a case fatality rate of 6.4%.^1^ The world is locked down; most businesses are closed; and people are keeping a social distance from each other, isolating themselves, washing hands for 20 s, not touching their faces, wearing masks and avoiding hospitals. The elderly of the society and those with chronic diseases are at the highest risk of dying. Five weeks of lockdown with almost no cars on the roads and no selling of alcohol have dramatically reduced mortality and morbidity from road accidents, murders and assaults.^2^ All routine outpatient clinics, elective surgery, outreach support and continuous medical education meetings have stopped. Hospitals and clinics are preparing for the coronavirus disease 2019 (COVID-19) crises, expecting to be overwhelmed. Traditional roles have changed; for example, orthopaedic medical officers and ophthalmology specialists are screening people for acute respiratory symptoms, some joining nurses and community health workers. Clinical rotations have changed; for example, ‘1 month in theatre’ has become ‘1 week in emergency centre, followed by 1 week in theatre’. Student assignments and examinations have been deferred or cancelled. There is a complete relook at the way we teach and learn. The healthcare system is being redesigned.

## Disruption and innovation

While ‘disruption’ is a negative term, it creates an environment for innovation, which is a positive term, well-described in technological industries.^3^ A disruption can be defined as an event in which a substantial share of agents belonging to a system is disrupted, typically requiring new skills and creating a pressure to change the value generation models of an organisation.^3^ The questions in the ‘room’ reflected a cautious concern for an uncertain future:

- How will this crisis affect the registrar training programme, including my learning plans?
- How will I complete my research, particularly the fieldwork?
- How will the exams work?
- How will we do outreach and support?
- How will I finalise my quality improvement cycle implemented at the clinic in order to complete my Leadership and Management module?

Can this crisis be the opportunity to relook at how family physicians live out our expected roles? Particularly, moving from a hospital-centred to a community-centred environment, working in multi-professional teams, focusing on preventative health and lifestyle versus curative, technocentric medicine? While we need intensive care units and ventilators, do we not need to understand self-care, community health and public health in a better way? The clinical expert role has been well developed, while the roles of community advocate and collaborator have been less well developed.

## Roles of the family physician revisited

The roles of the family physician in South Africa and the contribution to district health services have been well described.^4,5^ Apart from clinical competence, the family physician needs to be a consultant, capacity-builder, clinical governance leader, champion of community-oriented primary care (COPC) and clinical trainer.^4,5^

**FIGURE 1:**
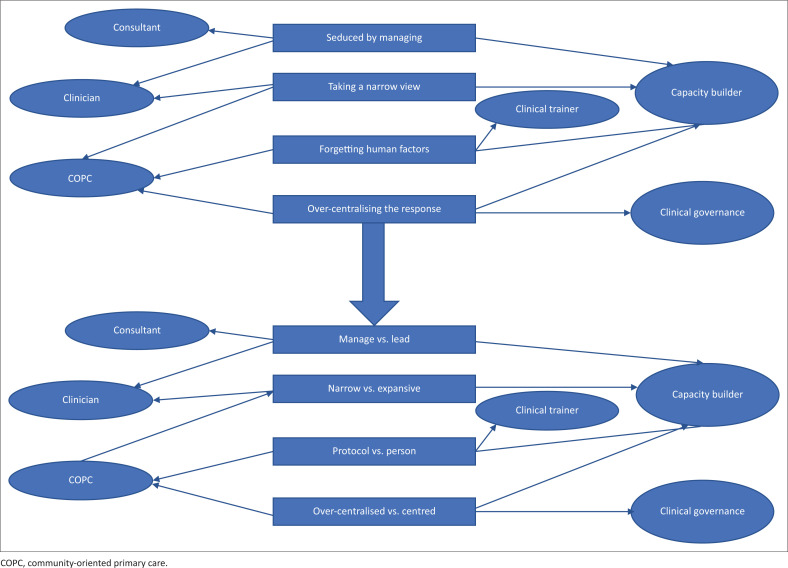
The roles of the family physician and potential leadership traps.

Researchers in a recent *Harvard Business Review* highlighted four traps that may prevent leaders in a crisis from balancing the management of the present with leading beyond the crisis.^6^ Family physicians, having a diverse and adaptive skill set, are regarded as expert generalists.^4^ Our roles make us leaders in our clinical and community contexts and, therefore, vulnerable to falling into these traps (see Figure 1). For example, over-centralising the response, in an attempt to control the crisis, can reduce capacity-building, missing an ideal opportunity to build capacity in others. In this example, our challenge is not to over-centralise, but remain ‘centred’ to our purpose and leadership role as family physicians. The solution is to adopt the cognitive aids depicted in Figure 1 (lower half).

Despite the roles of the family physician being broad, inclusive and adaptive, the COVID-19 crisis imposes challenges to exercise these roles. There are restrictions on movement, social distancing, de-escalation of elective procedures and minimisation of patient–clinician contact. Time for regular activities is reduced as we are called upon to assist in many contexts and capacities, from training cleaners to advising joint operations committees (JOC). We therefore need to use every opportunity to exercise our roles.

**FIGURE 2:**
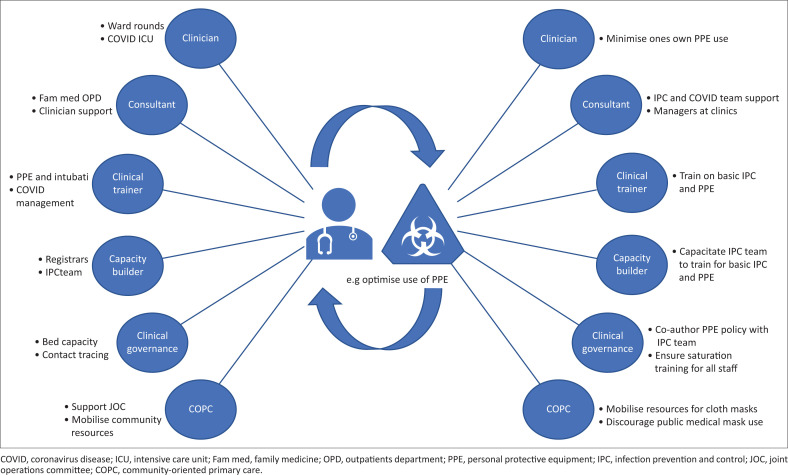
Traditional exercising of roles versus adapting to new activities.

Figure 2 (left side) presents the ‘traditional’ model of how a family physician might exercise his or her roles during the COVID-19 crisis. In contrast, the right side presents an example of an activity that the family physician is called upon to assist with, in this case, optimising personal protective equipment (PPE) use. This shows how the various ‘roles’ can be exercised from this one activity. The idea is to facilitate family physicians in exercising roles through *activity* rather than through *ourselves*. This enables agility and responsiveness during the crisis but still enables the expert generalist to use this crisis as an opportunity to strengthen the health system.

## A shift to a ‘new normal’

Coronavirus disease 2019 has made us realise how important understanding human behaviour is, something we still have not figured out with the tuberculosis epidemic.^7^ It has created a pressure to change our focus from routine activities to thinking critically about real health needs of individuals in communities, as well as staff health and safety. We need to innovate to prevent infection, promote health (including mental health), work closer with many more sectors of society, including social services, community volunteers, business sector, manage resources better and cooperate with the private health sector. A ‘new normal’ is setting in.^8^ A shift is happening from reactive curative care towards a more promotive, preventative community-based approach. Perhaps this is the time to shift from just measuring the leading causes of death to also include the leading causes of life.^9^ Health workers as well as people in communities need a sense of agency (power to do), to have hope (believe in solutions), to connect more (with self, others and environment) and to appreciate intergenerativity (realising that everyone is part of a continuum of those that went before and those that are coming after).^9^ Family physicians, with their skills set of clinical competence across all clinical disciplines, leadership skills of communication and collaboration within teams and viewing patients as part of families in communities, are ideally placed to respond appropriately to this crisis.

## Conclusion

The roles of the family physician have been disrupted by an invisible enemy. Preventative care in a patient-centred, community-oriented approach is of critical importance, now more than ever. The governance of resources, such as masks, gloves and medicines, has global implications. The isolation, uncertainty and fears of patients and colleagues necessitate diligent self-care, mental healthcare and a renewed focus on caring for each other. The skills to reflect, communicate and share within our teams are being sharpened like never before.
